# Pseudotumor of the Hip due to Fungal Prosthetic Joint Infection

**DOI:** 10.1155/2013/502728

**Published:** 2013-12-03

**Authors:** Stefano Artiaco, Andrea Ferrero, Frediano Boggio, Giulia Colzani

**Affiliations:** ^1^Department of Orthopaedics and Traumatology, Orthopaedic and Trauma Center, Via Zuretti 29, 10126 Turin, Italy; ^2^Department of Orthopaedics and Traumatology, Ospedale Regionale Bellinzona e Valli, Viale Officina 3, 6500 Bellinzona, Switzerland

## Abstract

Pseudotumors associated with total hip arthroplasty have been associated with metal-on-metal and metal-on-polyethylene total hip arthroplasties due to a granulomatous foreign-body reaction to methyl methacrylate, polyethylene, or metal ion release, but they have not been related to prosthetic joint infections. In this paper, we report an unusual case of *Candida albicans* total hip arthroplasty infection, causing a large inflammatory pseudotumor of the hip joint. Fungal periprosthetic joint infections are a rare clinical entity and difficult to diagnose, and a pseudotumor may be part of their clinical presentation. They should be suspected in immunodeficient host patients when clinical symptoms of prosthetic joint infections are observed.

## 1. Introduction

Despite surgical advances and antibiotics evolution, periprosthetic infections still represent a challenge for orthopaedic surgeons because of the demanding surgical treatment and unpredictable clinical results.

The most common pathogens involved in prosthetic joint infections are gram-positive *Staphylococcal* species bacteria followed by aerobic gram-negative *Bacilli* and *Anaerobes* species [1]. Fungal periprosthetic hip infections are rare, and clear and accepted treatment guidelines have not yet been established. Few case reports and limited clinical series have been published in the literature about this topic, and from the analysis of the available data only 21 patients with total hip arthroplasty, who were affected by fungal infections of joint prostheses, have been detected by Azzam et al. until 2009 [2]. *Candida albicans* was recognised as the responsible pathogen in 11 cases; others included *Candida* species such as *Parapsilosis*, *Glabrata*, and *Tropicalis* in 10 cases. Recently, 4 further cases of fungal periprosthetic hip infection due to *Candida* species pathogens have been reported by Anagnostakos et al. [3]. Fungal pathogens, other than *Candida*, have been reported only in a case of periprosthetic hip infection caused by Rhodotorula minuta [4].

In this paper, we report an atypical case of *Candida albicans* total hip arthroplasty infection, causing an inflammatory pseudotumor of the hip joint. To our knowledge, this is the first case reported in the literature in which such kind of clinical finding was identified in periprosthetic fungal infection of the joints.

## 2. Case Presentation

In 2010, a 70-year-old Caucasian woman was referred by an infectious disease specialist to the Bone and Joint Infection Unit of our Orthopaedic Department because of a *Candida albicans* periprosthetic infection of the left hip joint. This was confirmed by joint fluid cultures and laboratory tests. The patient was immunocompromised due to immuno-suppressive therapy with steroids and methotrexate, taken for rheumatoid arthritis and Sjogren's syndrome. Her orthopaedic history consisted of a total hip arthroplasty in 2004 and a subsequent early revision for prosthetic instability in the same year. In 2005, she was referred to our center for a coventry type II MRSA infection and underwent a two-stage revision total hip arthroplasty with an antibiotic-loaded cemented metal-on-polyethylene prosthetic implant. During the following years, she was free from infection having periodical ambulatory orthopaedic and infectious diseases checkup. However, at the last followup, the patient presented with a pain-free hip joint and a swelling of the greater trochanteric area, due to a pseudotumor mass containing a large amount of fluid which was aspirated under ultrasound guide. Cultures tests detected *Candida albicans* infection.

**Figure 1:**
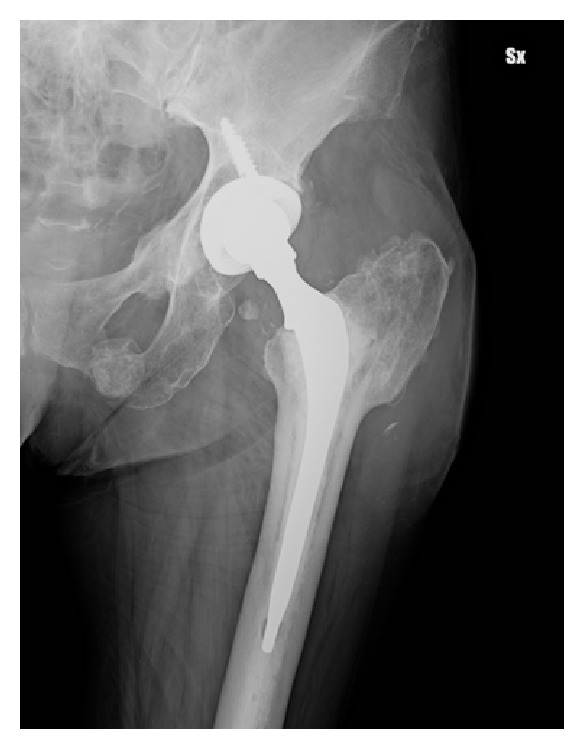
Preoperative X-ray total hip arthroplasty.

**Figure 2:**
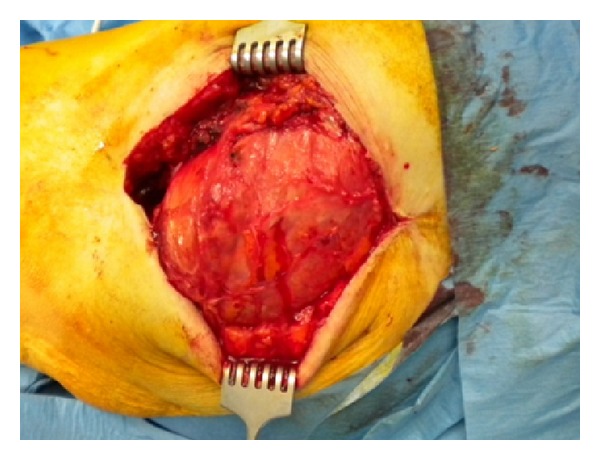
Intraoperative view of the left hip. Pseudotumor exposure.

**Figure 3:**
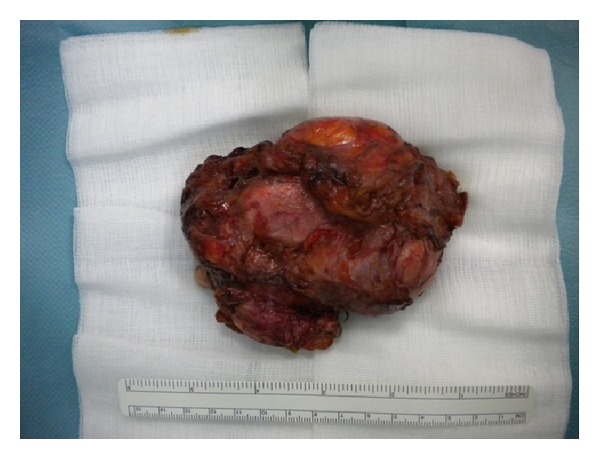
Pseudotumor specimen after surgical removal.

**Figure 4:**
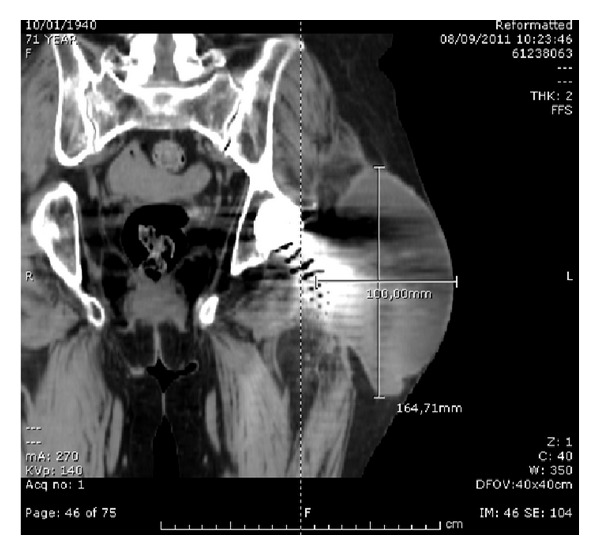
CT scan showing large septic joint fluid collection of the left hip.

At initial clinical exam in our department, the patient presented apyrexial, with a local swelling to the left hip but without redness or rise of local temperature. The range of joint motion of the hip was slightly limited but in line with a well-functioning prosthetic implant. Laboratory tests revealed increased levels of erythrocyte sedimentation rate (69 mm/sec) and C-reactive protein (34.1 mg/L). The patient was undergoing fluconazole therapy (200 mg/day) prescribed immediately after fungal infection detection. The radiographic exams did not show signs of periprosthetic bone resorption or implant loosening (Figure 1). The patient was informed about the complexity of pathology, and a two-stage prosthetic revision was offered, but due to absence of pain and limitations in daily life activities she refused this option accepting an attempt of treatment with debridement and irrigation. The operative procedure allowed the removal of the pseudotumor mass and drainage of the residual fluid abscess (Figures 2 and 3). A sample of septic fluid was taken for microbiological cultures and confirmed the growth of *Candida albicans*. Histological analysis demonstrated a large granulomatous cystic lesion (9 × 7 × 5 cm) containing purulent exudate. The antifungal therapy with fluconazole (200 mg/day) was continued for 6 months. The surgical site healed uneventfully in three weeks, and after surgical debridement and medical therapy the symptoms rapidly resolved. During the course of postoperative time, the patient did not complain of any symptoms and continued her normal daily activities. Nevertheless, 12 months after surgical debridement, she showed recurrence of hip swelling and a fluid joint effusion without pain or hip limitation (Figure 4). The aspirated fluid cultures were repeated and confirmed a persistent *Candida albicans* infection. The patient still refused two-stage revision arthroplasty, and only aspiration of the fluid was performed, continuing medical treatment by means of suppressive miconazole therapy.

## 3. Discussion

Pseudotumors associated with total hip arthroplasty are a relevant topic in current orthopaedic practice. Those lesions have been described over the last years, typically in patients with metal-on-metal total hip implants, and they have been related to foreign-body reaction, hypersensitivity, and wear debris [5]. They have also been reported in cemented and cementless metal-on-polyethylene total hip arthroplasty due to a granulomatous foreign-body reaction to methylmethacrylate, polyethylene, or metal [6]. Pseudotumors may cause pain and discomfort of a hip joint or symptoms related to compression of anatomical structures surrounding the expanding mass, and sometimes they may mimic an infection [5]. Pseudotumor with superimposed periprosthetic infection has been described as a case report following metal-on-metal hip arthroplasty [7], but, until now, no study correlated the growth of hip pseudotumor with periprosthetic fungal infection.

At present, to our knowledge, the case presented in this paper is the first reported in the literature, in which a pseudotumor developed in a patient with a fungal infection of hip prosthetic implant, caused by *Candida albicans*. In this case, the patient refused two-stage revision, which was the correct treatment for this rare infection, accepting only debridement and irrigation of the prosthetic hip implant [2, 8].

Most patients affected by candidal periprosthetic infections have comorbidities or risk factors which can impair immunity defense, including cardiac and hepatic diseases, prolonged antibiotic or steroids therapy, diabetes mellitus, and rheumatoid arthritis. Identification of risk factors may help in detecting this rare condition, which is confirmed by means of joint fluid cultures and laboratory tests. Usually, *Candida* prosthetic joint infection appears as a chronic infection during the late postoperative period, with pain, swelling, or drainage of the affected joint [9].

The clinical history of our case demonstrated that in an immunodeficient host with total hip arthroplasty a fungal infection should be suspected even when minor symptoms of joint sepsis are present and that the development of an infection-related pseudotumor may be observed in such kind of sepsis.
